# Crossed versus conventional pseudophakic monovision for high myopic eyes: a prospective, randomized pilot study

**DOI:** 10.1186/s12886-020-01694-5

**Published:** 2020-11-16

**Authors:** Yan Xun, Wenjuan Wan, Lu Jiang, Ke Hu

**Affiliations:** Department of Ophthalmology, The First Affiliated Hospital of Chongqing Medical University, No.1 Youyi Road, Yuzhong District, Chongqing, 400000 P.R. China

**Keywords:** Monovision, High myopia, Cataract, Spectacle independence, Vision quality

## Abstract

**Background:**

Aiming at spectacle independence, conventional pseudophakic monovision has been widely used in myopia patients with bilateral monofocal intraocular lens implantation. However, the crossed monovision, which is to correct the dominant eye for near vision and the non-dominant eye for distant vision, has been mentioned preferable for high myopic cataract patients by some studies.

We have conducted this study to compare clinical results to assess the feasibility of conventional and crossed monovision for high myopic pseudophakic patients by comparing patient satisfaction, visual function and spectacle independence.

**Method:**

Forty-sixth high myopia patients were divided into two groups: 22 in crossed monovision group with patients whose refraction targeted to − 2.00 diopters (D) in the dominant eye and − 0.50D in the non-dominant eye; 24 in conventional monovision group with patients whose refraction targeted to − 0.50D in the dominant eye and − 2.00D in the non-dominant eye. Binocular uncorrected distance visual acuity (BUDVA), binocular uncorrected near visual acuity (BUNVA), binocular corrected distant visual acuity (BCDVA), binocular corrected near visual acuity (BCNVA), contrast visual acuity and stereoacuity were examined at postoperative 2 weeks, 1 month and 3 months. Questionnaires were completed by patients 3 months after binocular surgery to evaluate patients’ satisfaction and spectacle independence.

**Results:**

The conventional monovision and the crossed monovision group showed no significant differences of mean BUDVA, BUNVA, BCDVA, BCNVA 2 weeks, 1 month or 3 months postoperatively (*P* > 0.05). There was no difference in the bilateral contrast sensitivity or stereoscopic function between the convention conventional and crossed monovision groups (*P* > 0.05). Patient satisfaction with near and distant vision, as well as spectacle dependence did not differ significantly between the two groups (*P* > 0.05).

**Conclusion:**

Crossed pseudophakic monovision exhibited similar visual function when compared with conventional monovision technique, which indicates that it is an effective option to improve the visual functionality and quality of life for high myopic patients who considering bilateral cataract surgery.

**Trial registration:**

The Institutional Review Board and Ethics committee of the First Affiliated Hospital of Chongqing Medical University, Chongqing, China. The trial registration was submitted in September 2018 and passed on March 18, 2020, and the registration number is: ChiCTR2000030935.

## Background

Due to the high incidence of high myopia [1], cataract patients with high myopia are also numerous. For these patients, cataract surgery provides an opportunity to ameliorate the refractive errors and improve the quality of binocular vision. Surgical design is particularly important for the prognosis of patients with high myopia. Optimal refractive target of intraocular lens (IOL) should be adapted to the living habits of preferred near vision for myopes [2]. Compared with normal population, high myopia patients are often associated with larger corneal astigmatism [3–5], as well as higher incidence of retinal detachment, tear, macular hemorrhage and neovascularization [6, 7]. These factors may limit the use of multifocal intraocular lens (IOL) in cataract patients with high myopia [8–13]. In addition, higher expenses, possible postoperative glare and long adaptation period caused by multifocal intraocular lens should also be carefully considered [14]. It has always been a challenge for cataract surgeons to satisfy patients with high myopia under complicated conditions.

Pseudophakic monovision was one of the methods to resolve the postoperative presbyopia, which was firstly applied in cornea refractive surgery, and has been widely used in myopia patients with bilateral monofocal intraocular lens implantation [15–19]. By making the refraction targets to distant for one eye and to near for another one, the patients would have both clear distant and near vision. The conventional monovision was set to correct the dominant eye for distance vision, and the non-dominant eye for near, which might be based on the hypothesis that it was easier to suppress the blur in non-dominant eye than in the dominant one [20–22]. However, other studies evaluated the opposite way of design, which is to correct the dominant eye for near vision and the non-dominant eye for distant vision, named crossed monovision [21, 22]. And the eye dominance may play an important role in the monovision design [23]. At present, there is little effective proof as to which method is more beneficial for high myopic cataract patients. We conducted this study to compare clinical results including vision function, patient satisfaction and spectacle independence between conventional and crossed pseudophakic monovision patients and evaluate which design is the better choice for high myopic cataract patients intending to have bilateral monofocal IOL implantation.

## Methods

### Patients

This study was a prospective randomized comparative study. Patients with myopic diopter greater than − 6.0D (including − 6.0D), axial length greater than 26.00 mm (including 26.00 mm), and lens opacification that affect life quality, who had strong desire of spectacle independence have been included in this study. All the subjects underwent bilateral cataract surgery at the First Affiliated Hospital of Chongqing Medical University from November 2018 to September 2019. This research adhered to the tenets of the Declaration of Helsinki and Uniform Requirements for manuscripts submitted to Biomedical journals.

### Exclusion

The exclusion criteria included any other ocular pathological conditions that would affect the visual acuity after surgery, such as dysfunction of optic nerve, macula, retina or cornea; severe ocular opaque other than cataract; history of non-cataract ocular surgery or inflammation; eyes planned for extracapsular cataract extraction; intractable synechia of iris; strabismus; individuals who cannot identify the dominant eye before surgery; patients refusing to be treated or having difficulty for follow-up examination.

### Randomization

Severe anisometropia (greater than 3.0D) may decrease stereoscopic function, thus insufficient unaided reading capacity also leads to dissatisfaction [18]. We assigned all the subjects randomly to one of the two groups before surgery. Based on the results of previous studies [21, 22, 24] and clinical experience, the design used in this study was about 1.5D anisometropia between both eyes to provide relatively sufficient near visual ability while avoiding losing the tolerance of stereopsis [20, 24]: the crossed monovision group was assigned with patients whose refraction was targeted to − 2.00D in the dominant eye and − 0.50D in the non-dominant eye, and the conventional monovision group with patients whose refraction was targeted to − 0.50D in the dominant eye and − 2.00D in the non-dominant eye.

The dominant eye was determined by the hole-in-card test in which each patient was asked to look at a fixed target at 2 m through a 3 cm diameter hole in a center of a cardboard held in their hands. The eyes were then covered one at a time and the eye that kept the alignment was recorded as the dominant eye. The test was repeated three times to confirm the dominant eye.

This was a pilot prospective, randomized clinical trial. Randomization was carried out according to simple randomization with randomized table. For each group, 25 participants were planned to randomly assigned, received intended treatment, and were analyzed for the primary outcome. The coordinate staff assigned each patient randomly to one of the two groups. The grouping information was passed by the coordinate staff to a member of the operating room to prepare the IOL. The surgeon performing the surgery was not informed to which group the patients belonged. Neither the patients, examiners, nor data analysts knew the group assignment.

Proceeded from the principle of double blindness, neither the surgeons nor the patients knew the specific grouping before and after operation. Before the surgery, the assistant staff had informed and helped the patient understand the design method of monovision and its possible advantages and disadvantages.

### Preoperative evaluation

Before operation, patients had a complete preoperative ophthalmic examination including binocular uncorrected distance visual acuity (BUDVA), binocular uncorrected near visual acuity (BUNVA), binocular best corrected distance visual acuity (BCDVA), binocular best corrected near visual acuity (BCNVA), subjective and objective refraction, biomicroscopy of the anterior and posterior eye segments, intraocular pressure (IOP), macular optical coherence tomography (OCT), keratometry, optical biometry. The IOLMaster 700 (Carl Zeiss Meditech AG, Jena, Germany) was used to measure preoperative axial length. Corneal curvature was measured using PENTACAM (PENTACAM®OCULUS, Germany), and the mean value of these meridians were used for the IOL power calculation. The IOL power was calculated using Haigis formula [25, 26].

### Surgical procedures

Standard phacoemulsification with IOL implantation was performed by one surgeon (KH M.D.). Under topical anesthesia, the corneoscleral incision was made at 135 degrees. After continuous circular capsulorhexis, cataract phacoemulsification and cortex aspiration were performed. The width of the main incision was 2.8 mm and the diameter of capsulorhexis was 5.5 mm. Then a monofocal aspheric posterior chamber IOL (Tesnis 1-piece ZCB00, Abbott Medical OPTICS, Inc) was implanted in the capsular bag.

Antibiotic, steriod and non-steroid anti-inflammatory eye drops were used 2 weeks after surgery.

### Main outcome measures

Examinations of visual function including BUDVA, BUNVA, BCDVA, BCNVA, and the manifest spherical equivalent value (MRSE) were conducted 2 weeks, 1 month and 3 months after binocular consecutive surgery. MRSEs were calculated using spherical and cylindrical powers (MRSE = sph + cyl/2). An application system (Zengshineng, Galen RS20YY-JT2005) of visual bio-informatics stimulation technology was used for assistant examination of the stereoscopic function [27, 28]. The stereoacuity at 0.8 m (near) and 1.5 m (distant) was categorized into three levels respectively as normal (100 arcsec), reduced (200–400 arcsec) and lack of stereopsis. The contrast visual acuity (contrast VA) was examined at high to low contrast levels using the contrast sensitivity tester (Vista Vision™ Clinica De Oftalmologie, Romania). A questionnaire based on the Visual Function Questionnaire 25 [29] was requested to complete 3 months after bilateral cataract surgery. On the questionnaire, patients were asked to rate their need for glasses or contact lenses after bilateral surgery from three levels (never need glasses or contact lenses, sometimes need glasses or contact lenses, always dependent on glasses or contact lenses). Patients were also asked other questions: rating their satisfaction from four levels (very happy, happy, neutral and unhappy); rating their eye-hand and eye-feet coordination without glasses (or contact lenses) from three levels (no problem, almost no and sometimes); rating their sports-related activities without glasses (or contact lenses) coordination from three levels (no problem, almost no and sometimes); rating their frequency of using glasses (or contact lenses) for near (within 0.4 m) / intermediate (1-3 m) / far (> 3 m) distance activities from three levels (never, sometimes and always). The results of the questionnaire were collected anonymously and recorded by face-to-face inquiry.

### Statistical analysis

Nonparametric tests were used for some data that do not follow the normal distribution. All the visual acuity was expressed as the logarithm of the minimum angle of resolution (logMAR). Continuous variables such as binocular uncorrected or corrected logMAR visual acuity (BUDVA, BUNVA, BCDVA, BCNVA), MRSE, and contrast visual acuity were compared between the conventional and crossed monovision groups, using the Mann-Whitney U test. Categorical variables such as the ratio of men to women and the grades of stereopsis were compared between the two groups using the χ^2^ test and Fisher exact test. Statistical analyses were performed using SPSS statistical software (version 22.0; SPSS Inc., Chicago, IL). The sample size was calculate based on 80% statistical test power, 0.05 significance level. A *P* value of less than 0.05 was considered statistically significant.

## Results

Fifty patients were enrolled and four were excluded. One case in the conventional group and three in the crossed group failed to participate in the follow-up on time and were excluded from the statistical analysis (24 in conventional monovision group, 22 in crossed monovision group were finally included). The mean age (±standard deviation [SD]) is 65.33 ± 10.87 years (range 39–85 years) in the conventional group, and 68.58 ± 11.56 years (range 39–85 years) in the crossed group. Baseline characteristics of both groups are shown in Table 1. Age, gender, axial length, MRSE, corneal astigmatism were of no statistically difference between the two groups (*P* > 0.05) (Table 1). The visual acuity (including BUDVA, BUNVA, BCDVA and BCNVA) of both groups was significantly improved compared with those before operation (*P* <0.05, Table 2).

**Table 1:** Preoperative Characteristics in Conventional Monovision Group and Crossed Monovision Group

Preoperative characteristics	Conventional Monovision Group	Crossed Monovision Group	*P* value
**Age (years)**	65 ± 11	69 ± 12	0.272
Sex (M/F)*	7/17	10/12	0.361
**Axial length (mm)**			
Distant eyes (Mean ± SD)	28.31 ± 2.27	29.17 ± 2.08	0.612
Near eyes (Mean ± SD)	28.40 ± 2.46	28.93 ± 2.40	0.489
**Corneal astigmatism (D)**			
Distant eyes (Mean ± SD)	−0.58 ± 1.32	−0.29 ± 1.17	0.311
Near eyes (Mean ± SD)	−0.56 ± 1.33	−0.72 ± 1.00	0.741
**MRSE (D)**			
Distant eyes (Mean ± SD)	−11.34 ± 5.87	−10.16 ± 6.26	0.652
Near eyes (Mean ± SD)	−12.53 ± 7.32	−10.03 ± 5.47	0.385
**Number of patients**	24	22	
Dominancy (R/L)*	18/6	14/8	0.525

Mann-Whitney test, *p* < 0.05

*χ2 test, *p* < 0.05

*M* Male, *F* Female, *SD* Standard deviation, *D* Diopters, *MRSE* Manifest spherical equivalent value

**Table 2:** Comparison of mean (± standard deviation) logarithm of the minimum angle of resolution (logMAR) Visual Acuity in Conventional Monovision Group and Crossed Monovision Group at postoperative 2 weeks, 1 month and 3 months

	Preoperative (Mean ± SD)			Postoperative 2 weeks (Mean ± SD)			Postoperative 1 month (Mean ± SD)			Postoperative 3 months (Mean ± SD)		
Visual Acuity (logMar)	Conventional Monovision Group	Crossed Monovision Group	*P*	Conventional Monovision Group	Crossed Monovision Group	*P*	Conventional Monovision Group	Crossed Monovision Group	*P*	Conventional Monovision Group	Crossed Monovision Group	*P*
BUDVA	0.90 ± 0.41	0.83 ± 0.35	0.823	0.25 ± 0.15	0.26 ± 0.22	0.761	0.20 ± 0.16	0.18 ± 0.13	0.854	0.20 ± 0.12	0.20 ± 0.15	0.573
BUNVA	0.72 ± 0.50	0.63 ± 0.27	0.156	0.26 ± 0.15	0.27 ± 0.12	0.829	0.20 ± 0.16	0.22 ± 0.13	0.434	0.27 ± 0.18	0.28 ± 0.25	0.576
BCDVA	0.43 ± 0.27	0.35 ± 0.23	0.451	0.08 ± 0.11	0.08 ± 0.14	0.831	0.05 ± 0.08	0.07 ± 0.07	0.251	0.07 ± 0.09	0.08 ± 0.12	0.792
BCNVA	0.38 ± 0.34	0.40 ± 0.22	0.345	0.14 ± 0.12	0.19 ± 0.15	0.283	0.12 ± 0.10	0.13 ± 0.10	0.538	0.15 ± 0.13	0.19 ± 0.12	0.263
**Number of patients**	24	22	/	24	22	/	24	22	/	24	22	/

Mann-Whitney test, *p* < 0.05

*LogMAR* Logarithm of the minimum angle of resolution, *SD* Standard deviation, *BUDVA* Binocular uncorrected distant visual acuity, *BUNVA* Binocular uncorrected near visual acuity

### Postoperative refraction

In the crossed monovision group, mean MRSE 3 months post-operation was − 1.76 ± 0.54D in the dominant eyes and − 0.31 ± 0.94D in the non-dominant eyes, while in the conventional monovision group mean refraction was − 0.39 ± 0.82D in the dominant eyes and − 1.72 ± 0.85D in the non-dominant eyes. The mean MRSE in the conventional monovision group was not statistically different as compared with that of the crossed monovision group on 2 weeks,1 month and 3 months follow-up after surgery (*P* > 0.05) (Table 3). However, the mean MRSE in eyes of near vision of the two groups did not reach the set level on 1 month or 3 month (Table 3), but not statistically different from that of 2 weeks post-operation (*P* > 0.05).

**Table 3:** Comparison of Postoperative Refraction in Distant eyes and Near eyes between Conventional Monovision Group and Crossed Monovision Group at postoperative 2 weeks, 1 month and 3 months

Postoperative MRSE (D)	Conventional Monovision Group	Crossed Monovision Group	*P* value
Postoperative 2 weeks	*N* = 24	*N* = 22	
Distant eyes (Mean ± SD)	−0.42 ± 0.69	−0.34 ± 0.64	0.473
Near eyes (Mean ± SD)	−1.91 ± 0.89	−1.79 ± 0.86	0.823
Postoperative 1 month	*N* = 24	*N* = 22	
Distant eyes (Mean ± SD)	−0.56 ± 0.65	−0.32 ± 0.79	0.355
Near eyes (Mean ± SD)	−1.77 ± 0.88	−1.87 ± 0.47	0.909
Postoperative 3 month	*N* = 24	*N* = 22	
Distant eyes (Mean ± SD)	−0.39 ± 0.82	−0.31 ± 0.94	0.690
Near eyes (Mean ± SD)	−1.72 ± 0.85	−1.76 ± 0.54	0.806

Mann-Whitney test, *p* < 0.05

*MRSE* Manifest spherical equivalent value, *D* Diopters, *SD* Standard deviation

### Distant and near binocular visual acuity

Binocular uncorrected and corrected visual acuity for both near (0.4 m) and far (5 m) distance (BUDVA, BUNVA, BCDVA, BCNVA) didn’t show statistical difference between the two groups at postoperative 2 weeks (*P* = 0.761 for BUDVA, *P* = 0.829 for BUNVA, *P* = 0.831 for BCDVA, and *P* = 0.283 for BCNVA), 1 months (*P* = 0.854 for BUDVA, *P* = 0.434 for BUNVA, *P* = 0.251 for BCDVA, and *P* = 0.538 for BCNVA) or 3 months (*P* = 0.573 for BUDVA, *P* = 0.576 for BUNVA, *P* = 0.792 for BCDVA and *P* = 0.263 for BCNVA). (Table 2).

### Binocular contrast visual acuity

**Fig. 1:**
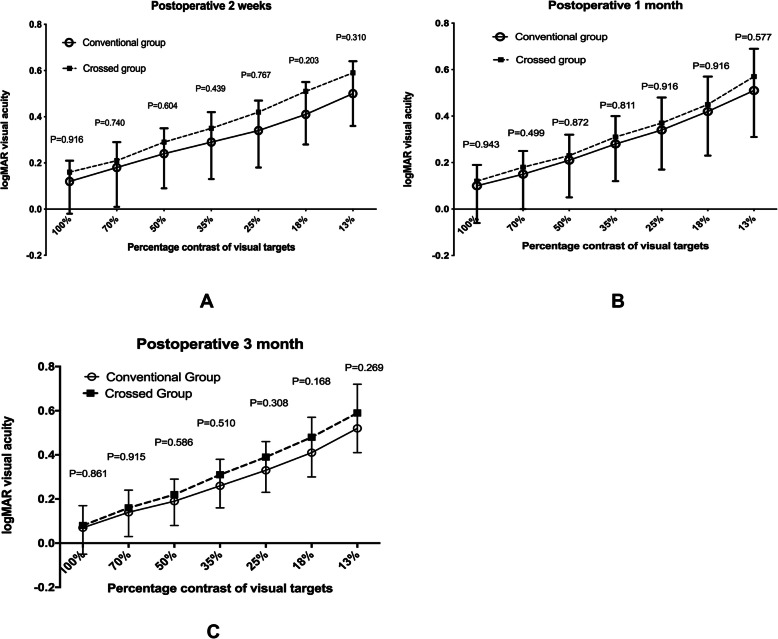
Comparison of postoperative binocular contrast sensitivity of seven different level of percentage contrast of visual targets (test distance: 3 m) between conventional monovision group and crossed monovision group at postoperative 2 weeks (**a**), 1 month (**b**) and 3 months (**c**)

The bilateral contrast visual acuity, which was converted into the logarithm of the minimum angle of resolution (logMAR) was better in conventional monovision group than that of crossed monovision group, however it had no statistical difference between the two groups (*P* > 0.05) at the seven level of contrast percentages of visual targets of postoperative 2 weeks, 1 month or 3 months (Fig. 1).

### Binocular stereoscopic function

**Fig. 2:**
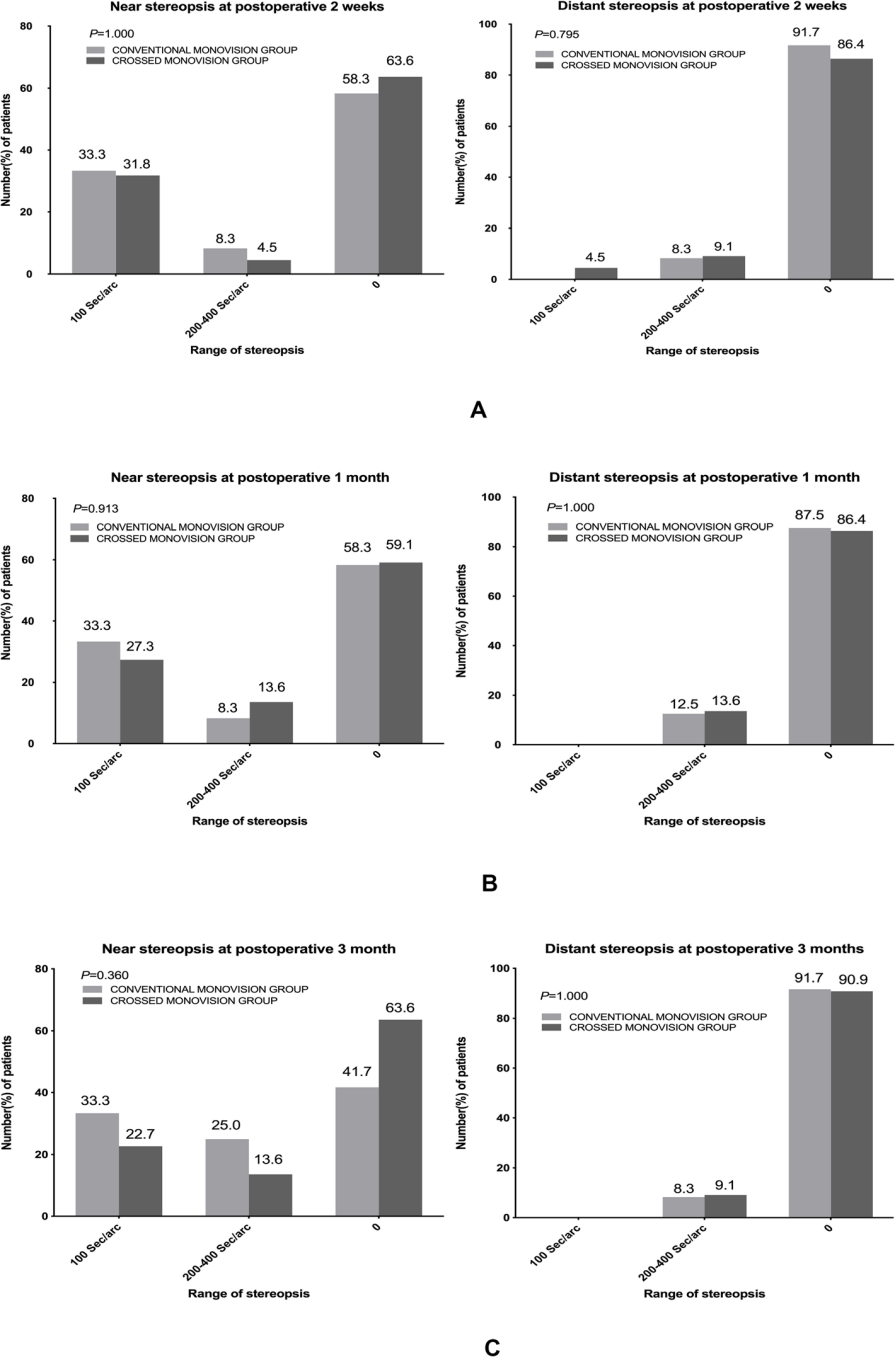
Comparison of postoperative near (0.8 m) and distant (1.5 m) stereopsis between conventional monovision group and crossed monovision group

The differences of binocular near stereopsis between the two groups were not statistically significant among the three checkpoints (*P* > 0.05, Fig. 2). Meanwhile, the results of distant stereopsis also showed no significant differences between the two groups (*P* > 0.05, Fig. 2).

### Spectacle independence; patient satisfaction; near, intermediate and distant vision performance

**Fig. 3:**
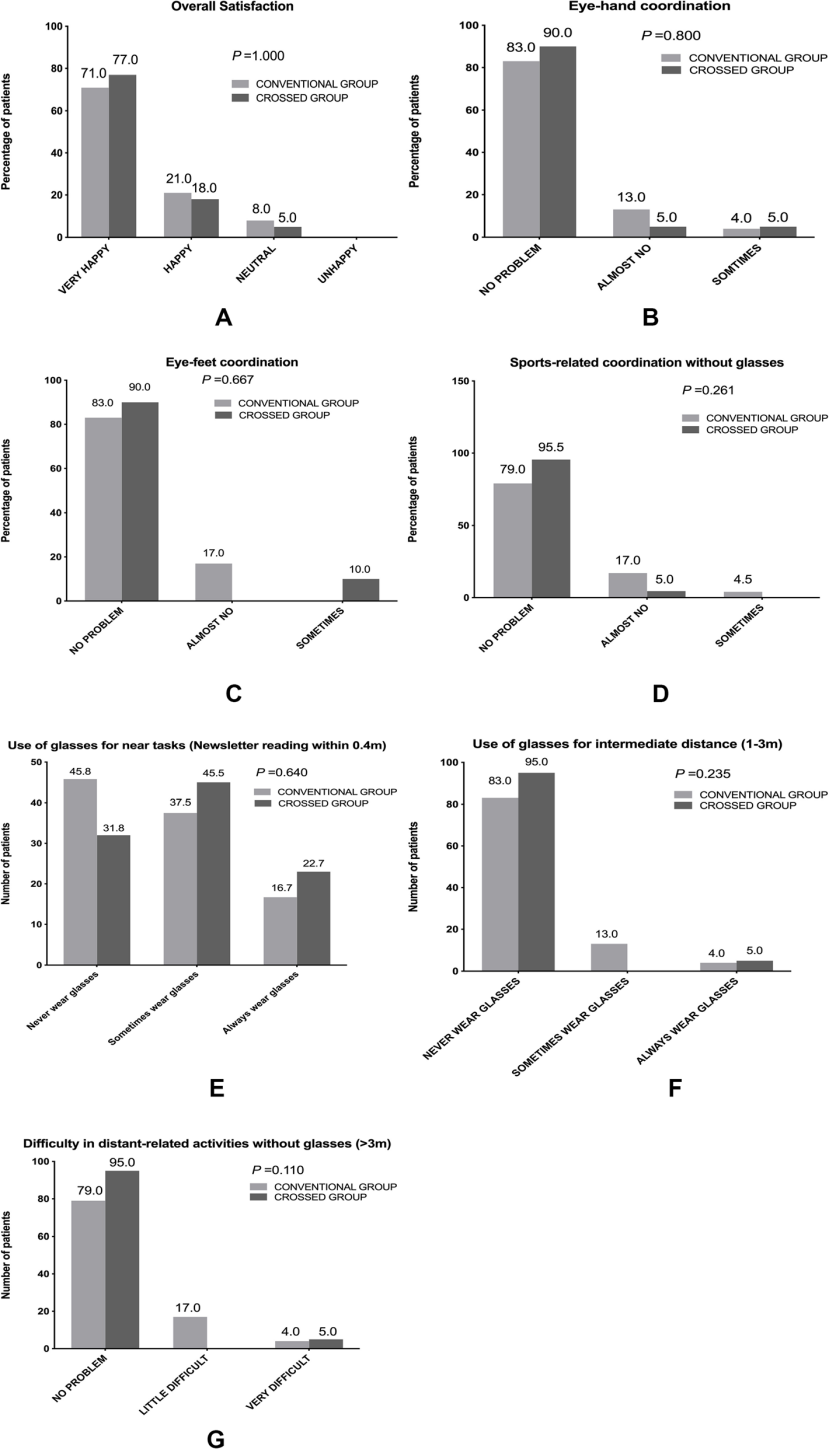
Comparison of postoperative overall satisfaction, Eye-hand coordination, Eye-foot coordination, Sports-related coordinations without glasses, Use of glasses for near tasks, Use of glasses for intermediate distance, and Difficulties in distant-related activities without glasses between conventional monovision group and crossed monovision group estimated by questionnaires

A total of 20 (83.3%) patients of the conventional group and 18 (81.8.0%) in the crossed group didn’t wear spectacles after cataract surgeries, which has no statistically significant difference between two groups (*P* = 1.000). Patients’ satisfaction, eye-hand and eye-feet coordination, sports-related coordination, difficulties for near (0.4 m) distance tasks without glasses (or contact lenses), difficulties for intermediate distance activities without glasses (or contact lenses) and difficulties for far (> 3 m) distance tasks without glasses (or contact lenses) didn’t differ significantly between the crossed and the conventional group 3 months after surgery (*P* > 0.05, Fig. 3).

### Changes of the eye dominance

After binocular surgery, we measured the eye dominance of all patients again at postoperative 2 weeks, 1 month and 3 month. No change of eye dominance from one to the other was found.

## Discussion

To improve the visual quality of high myopic cataract patients with spectacle independence has been a great challenge for cataract surgery, since the lack of management for postoperative presbyopia [16, 29, 30]. Our study determined that pseudophakic monovision was an effective and low-cost option for high myopic patients who would like to reduce dependence on spectacles postoperatively. And both conventional and crossed monovision design presented similiar postoperative visual function, patient satisfaction, as well as spectacle independence.

Either crossed or conventional pseudophakic monovision should be considered along with multifocal IOL in choices available for high myopic cataract patients to deal with presbyopia. The monovision designed to correct hyperopia was initiated from cornea refractive surgery [3], some of the studies have shown that whether conventional or crossed monovision was not significantly related to postoperative satisfaction [16, 30]. The early application of conventional pseudophakic monovision has been reported in 1984 [20]. As conventional monovision was widely used, different amount of anisometropia between near and distant eyes has also been studied and prvoved to be effective using moderate anisometropia (< 2.0D) in conventional pseudopakic monovision [24, 31–33], which was similar to our postoperative results. Nevertheless, some studies concluded that crossed monovision was also effective [34, 35], but has not been studied in patients with high myopia specifically. In our study, the distance and near visual function are significantly improved in both crossed and conventional monovision group. There was no discomfort due to anisometropia between the two groups. Stereoacuity and contrast sensitivity of follow-up checkpoints showed similar results between the two groups. The spectacle independence was similar between crossed and conventional monovision groups, the general postoperative satisfaction, eye-hand and eye-feet cooperation, difficulty for near tasks were also at the same level, which was consistent with previous researches [21].

It might be more reasonable to use crossed monovision design which corrected the dominant eye for near vision and the non-dominant one for distance in cataract surgeries, since the refractive status would be more similar to the physiological characteristics of both eyes. The preferential use of the dominant eye for viewing might render the dominant eye more myopic than the non-dominant eye [36]. The dominant eye has a greater degree of myopia than the non-dominant eye, which explained the relationship between better visual acuity and ocular dominance in myopia patients [36]. Individual with strong rivalry dominance might have more difficulty suppressing the blur [37], and the success and satisfaction in pseduphakic monovision patients were significantly influenced by the magnitude of ocular dominance [38].

However the binocular visual outcomes of crossed monovision group are not better than conventional group as predicted. One possible reason might be that the anisometropia set both for crossed monovision and conventional monovision seemed not great enough to break the binocular balance. Another explanation might be that the magnitude of ocular dominance was not that strong in high myopic cataract patients. Seijas O et al. studied the response varying between different ocular dominance tests, and concluded that no clear ocular dominance was found in most studied subjects. They inferred that most patients who had been well tolerated for the establishment of monovision due to a continuous alternating balance between the eyes [39]. The adult brain may have some degree of plasticity throughout life. If one eye was patched for 150 min, the eye dominance in adults changed to some extent [40, 41]. Zhou and his colleagues reported that eye dominance could be adjusted in real time when viewing natural images [42].

It was worth mentioning that for certain myopia patients with cataract, it might not be able to accurately confirm the dominant eye before operation which will cause the unexpected binocular monovision. Either crossed or conventional monovision design would be optimal in most of these patients according to our results. Reasonable communication and proper training post operation would be strongly recommended to the adaption of monovision. Nevertheless, there were no individuals who had ever worn contact lenses in this study. If there are related subjects in future studies, some of these patients might have been in monovision contact for some years before surgeries. We should measure the dominant eye with and without corneal contact lens in those patients with less severe cataract, and use lens to simulate the refractive state before operation to achieve the patient’s satisfaction.

In addition, some studies concluded that a history of external ocular muscle surgery, apparent tropia and phoria, and a history of chronic imbalance would be the potential contraindications to IOL monovision, especially crossed monovision [21]. A highly demanding personality would possibly make the patient unwilling to cooperate [21]. So the surgical design should be considered carefully according to the individual situation.

The insufficiency of the study included the relatively small sample size. The subjects were all cataract patients with axial length longer than 26 mm, which will inevitably bring challenges to the preoperative biometric measurement. Studies with enlarged sample size, prolonged follow-up time, and more comprehensive observation should be conducted in the future.

## Conclusion

In conclusion, both conventional and crossed monovision design are effective to reduce dependence on spectacles after monofocal IOLs implantation for high myopic cataract patients, which improve patients’ quality of life and elevates both distant and near vision without differences. Therefore, conventional or cross-monocular vision can be recommended for high myopic cataract patients.

Eye dominance (ED) is known as a complex property including several types [43]. The most commonly established types of ED include the sighting dominant eye, which refers to the eye preferentially used when performing a monocular task, as well as the sensory dominant eye which is defined as the eye whose perception is stronger during binocular rivalry [44]. And the most used and less variable ED was sighting ED [45]. One of the most favorite way to assess sighting ED was the “hole in-card-test” [45], which had been proved to have great test-retest reliability [43, 44, 46–48]. Considering that the ED might have been changed in some patients after bilateral cataract surgery and to know more about the impact of ED exchange on postoperative quality of vision, we have considered the eye dominance 2 weeks, 1 month and 3 months after bilateral surgery using the same hole-in-card method. The result showed no changes of dominant eye had happened among all the tested patients. Further clinical research about the eye dominance changes would be needed.

## Data Availability

The datasets used and/or analysed during the current study are available from the corresponding author on reasonable request.

## Abbreviations

IOL: Intraocular Lens
D: Diopter
OCT: Optical Coherence Tomography
IOP: Intraocular Pressure
BUDVA: Binocular Uncorrected Distant Visual Acuity
BUNVA: Binocular Uncorrected Near Visual Acuity
BCDVA: Binocular Corrected Distant Visual Acuity
BCNVA: Binocular Corrected Near Visual Acuity
MRSE: Manifest Spherical Equivalent Value
Sph: Spherical Diopters
Cyl: Cylinder Diopters
logMAR: Logarithm of the Minimum Angle of Resolution
ED: Eye Dominance
